# Polymorphic Ventricular Tachycardia in Acute Cervical Spinal Cord Injury: A Rare Occurrence in the Setting of Normal QTc

**DOI:** 10.7759/cureus.53299

**Published:** 2024-01-31

**Authors:** Dibya S Mahanta, Anup K Budhia, Rama C Barik, Debasish Das, Ranjan K Mohanty, Debasis Acharya

**Affiliations:** 1 Cardiology, Institute of Medical Sciences and Sum Hospital, Bhubaneswar, IND; 2 Internal Medicine, Hi-Tech Medical College and Hospital, Bhubaneswar, IND; 3 Cardiology, All India Institute of Medical Sciences, Bhubaneswar, Bhubaneswar, IND; 4 Cardiology, SCB Medical College & Hospital, Cuttack, IND

**Keywords:** neurogenic shock, autonomic dysreflexia, qtc interval, spinal cord injury, polymorphic ventricular tachycardia

## Abstract

Cardiovascular manifestations like bradycardia, hypotension, fluctuation of blood pressure, and supraventricular arrhythmia are common in acute spinal cervical injury above the C6 level and are the major cause of mortality and morbidity in them. Ventricular tachycardia (VT) and fibrillation have only been reported in a few cases, but polymorphic VT (PMVT) has not been reported. We report a very rare case of acute cervical spinal cord injury patient who developed PMVT in the setting of normal QT interval degenerating to ventricular fibrillation, causing cardiac arrest before surgery.

## Introduction

Spinal cord injury is common, and cardiovascular system involvement in acute and chronic phases is the primary cause of morbidity and mortality in those affected by cervical spinal cord injury above T6 [1]. Major cardiovascular manifestations include bradycardia, supraventricular tachycardia, hypotension, fluctuation of blood pressure and heart rate, and, rarely, cardiac arrest [2]. Ventricular tachycardia (VT) and fibrillation have only been reported in a few cases, but polymorphic VT (PMVT) has not been reported [3]. Clinicians need to be cognizant of this potential arrhythmic complication and consider close cardiac monitoring, especially in the initial phase post-injury.

This article was previously posted in Authorea (January 18, 2021, found at https://doi.org/10.22541/au.161097943.34107135/v1), but it has not been submitted or published yet in any journal.

## Case presentation

**Figure 1:**
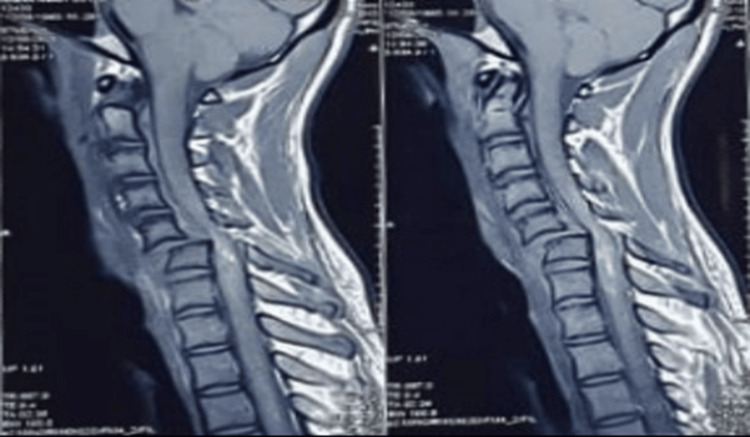
MRI of cervical spine Cervical spine MRI showing the fracture dislocation at the level C5 and C6 vertebra with compression to the spinal cord at that level.

**Figure 2:**
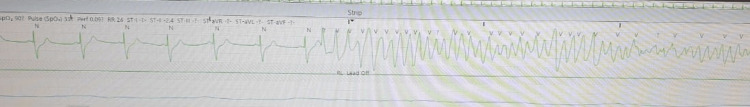
ECG during VT episode Normal QTc interval during sinus rhythm, R on T phenomenon followed by initiation of PMVT and later degenerating to ventricular fibrillation.

**Figure 3:**
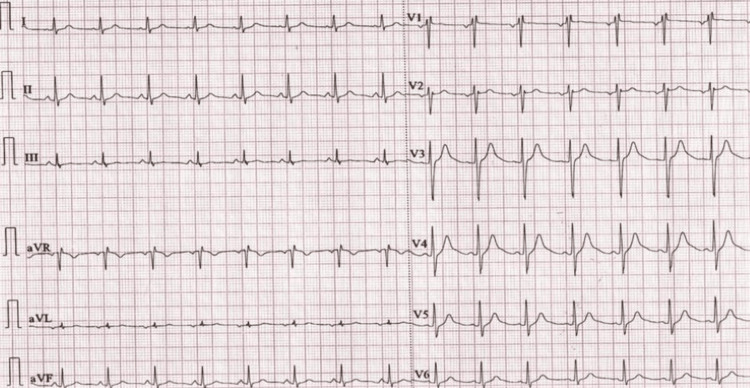
ECG one hour before the event Sinus rhythm, heart rate of around 100 per minute, and a normal QTc 448 msec.

**Figure 4:**
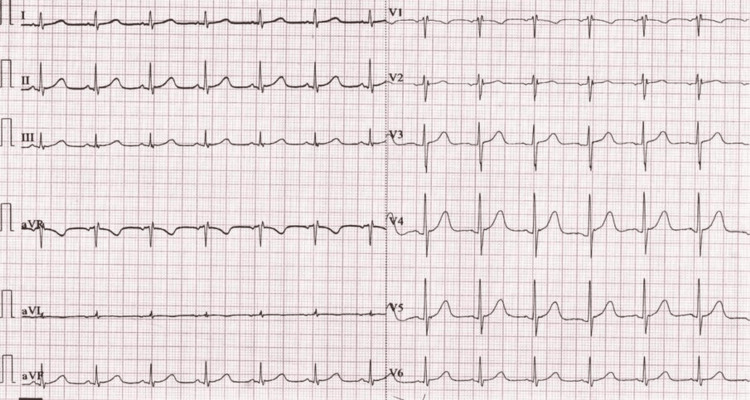
ECG just after the event Sinus rhythm with QTc 445 msec, no ST/T changes.

A 25-year-old male presented at the emergency department one hour after a road traffic accident. He had no past or family history of any significant illness. Upon examination, his supine blood pressure was 110/78 mm Hg in the right arm, and his pulse rate was 90 bpm. The nervous system examination revealed complete flaccid quadriplegia. He had type 1 respiratory failure due to diaphragmatic palsy and was immediately intubated and mechanically ventilated. He was shifted to the radiology department for emergency spine imaging. MRI of the cervical spine with a screening of the whole spine revealed fracture dislocation of the cervical vertebra at C5 and C6 levels with local impingement and compression of the spinal cord (Figure 1). Given an acute spinal cord injury presenting quadriparesis with diaphragmatic palsy, the decision was taken for emergency spinal cord decompression and fixation of the fractured and dislocated cervical vertebra. All routine blood tests, such as complete blood counts (CBC), renal function tests (RFT), liver function tests (LFT), and electrolytes, were within normal limits (total leucocyte count of 7,840 cells/μl, hemoglobin (Hb) of 13.8 g/dL, blood glucose of 97 mg/dL, calcium of 8.9 mg/dL, sodium of 139 mEq/L, potassium of 4.4 mEq/L, bicarbonate of 26 mEq/L, blood urea nitrogen of 19 mg/dL, and creatinine of 0.98 mg/dL). Arterial blood gas analysis (ABG) at the time of admission to the emergency room showed pH 7.46, PaO2 (partial pressure of oxygen) of 50 mm Hg, PaCO2 (partial pressure of carbon dioxide) of 35 mm Hg, HCO3 (bicarbonate) of 24 mEq/L, and O2 saturation (SaO2) of 88%, findings consistent with type 1 respiratory failure. PaO2 and O2 saturation and pH improved and became normal two hours after intubation and mechanical ventilation. In the preoperative room, he developed sudden PMVT (Figure 2), degenerating into ventricular fibrillation and cardiac arrest. Immediately, cardiopulmonary resuscitation was started, and the patient was revived. An intravenous lidocaine bolus of 1 mg/kg was administered, followed by an intravenous infusion of 1 gram of magnesium sulfate over 30 minutes. Serum electrolytes, including serum sodium (Na+), potassium (K+), magnesium (Mg++), and calcium (Ca++) just after the event, were within normal limits. No significant bradycardia episode was noted on the monitor before the arrhythmic event. ECG before and after the episode of PMVT revealed incomplete right bundle branch block (RBBB), normal QTc (corrected QT interval) intervals of 448 msec and 445 msec, respectively, and no ST/T wave abnormality (Figures 3-4). An ECG showed a structurally normal heart, no wall motion abnormalities, and no pericardial effusion with good biventricular function. All medications used for the patients were reviewed, and no QTc-prolonging medication was found. Although the patient had no cardiovascular risk factors, a coronary angiogram (CAG) was done just after the arrhythmic event to rule out ischemic causes of PMVT, revealing normal coronaries. The patient was taken for cervical spinal cord decompression and fixation of fractured vertebrae. Subsequent ECGs done in the patient during the hospital stay revealed the QTc interval to be in the normal range.

As the patient had a life-threatening arrhythmia and had incomplete RBBB in baseline and subsequent ECG, we performed a Brugada challenge test using oral flecainide, which came out to be negative. Approximately 72 hours of Holter monitoring revealed no intermittent QTc prolongation, malignant ventricular premature beats (VPC), or arrhythmia. An electrophysiology study (EP study) was done, and no arrhythmia, accessory pathway, or sinus node dysfunction was noted. Cardiac MRI (CMRI) did not reveal any structural abnormalities. He was given the option of an implantable cardioverter defibrillator (ICD) implantation. Gradually, the patient completely recovered from quadriparesis and was discharged in stable condition after ten days. On follow-up after three months, he was subjected to provocative tests like the epinephrine challenge test (Mayo protocol), adenosine challenge test, and an exercise test to rule out congenital long QT syndrome and catecholaminergic PMVT (CPMVT), all of which came out to be negative. He had no presyncope, syncope, or palpitation symptoms at six months of follow-up.

## Discussion

Spinal cord injury is common and can lead to severe neurological sequelae if untreated. Cardiovascular system involvement is the primary cause of morbidity and mortality in cervical spinal cord injury patients [1]. Cardiovascular dysfunction is the most common cause of death in spinal cord injuries, accounting for approximately 30% of all-cause mortality [1]. Disruption of the sympathetic system and unopposed vagal stimulation from spinal cord injury above the T6 vertebra level can have various cardiovascular effects. Significant cardiovascular effects are persistent bradycardia, neurogenic shock, rarely asystole, and cardiac arrest. Some patients may develop paroxysmal supraventricular tachycardia, atrial flutter, and fibrillation [3]. Patients with partial spinal cord injury may experience autonomic dysreflexia, which is characterized by a sudden increase in blood pressure because of sensory stimulation. The most common dysrhythmia in acute cervical cord injury is persistent sinus bradycardia, which peaks at day four and generally resolves by four to six weeks [4]. A few cases of VT and ventricular fibrillation have been described in patients with spinal cord injury in the literature [5]. It may develop if the patient has certain risk factors like electrolyte imbalance, QT-prolonging drugs, previous cardiac disease, or if the patient develops stress-induced cardiomyopathy [6]. However, PMVT has not been reported yet. Our patient had no history of any significant cardiac illness, had a structurally normal heart during the event and at discharge, and was not on any QT-prolonging drugs. All serum electrolytes were within normal limits.

PMVT can occur in the setting of a normal or increased QT interval. PMVT in the setting of a normal QT interval occurs in acute myocardial ischemia, Brugada syndrome, CPMVT, digitalis toxicity, and hypertrophic cardiomyopathy. PMVT in the setting of an increased QT interval is known as torsade de pointes (TdP) and can be congenital or acquired. Acquired long QTc is mainly because of electrolyte imbalances and QT-prolonging drugs. This distinction is important because treatment options for both categories are quite different. Interestingly, PMVT in acute cervical cord injury degenerating to ventricular fibrillation and cardiac arrest has not been described in the literature so far. Our case is unique and the first to describe PMVT in an acute cervical cord injury, which was successfully managed. PMVT with a normal QT interval can be treated with lidocaine or amiodarone, whereas amiodarone is contraindicated in PMVT with a prolonged QT interval. Intravenous magnesium sulfate, isoprenaline infusion, and rapid pacing at 100-120 per minute with a temporary pacemaker to decrease QT interval are treatment options in PMVT with prolonged QT or torsade de pointes. In our case, the triggering event for PMVT may be an acute autonomic imbalance in spinal cord injury. Studies have shown that low heart rate variability (HRV) and high QT dispersion index are associated with arrhythmogenicity, and both parameters are altered in high cervical spinal cord injury [3,4].

## Conclusions

Our case is unique and the first to describe the occurrence of PMVT in acute spinal cord injury, which has not been described in the literature so far. However, people have described spontaneous development of ventricular fibrillation and cardiac arrest amounting to cardiopulmonary resuscitation in acute spinal cord injury. A sympathovagal imbalance in acute spinal cord injury may be attributable to PMVT development in acute spinal cord injury, which needs utmost therapeutic management to rescue the patient. Critical care physicians in the trauma and emergency departments must be vigilant enough to recognize this form of tachycardia. It is crucial to distinguish between PMVT with normal QT and PMVT with QT prolongation. Treating the second type with conventional amiodarone can prolong the QT even more and lead to irreversible cardiac arrest.
